# Pectinase Activity Determination: An Early Deceleration in the Release of Reducing Sugars Throws a Spanner in the Works!

**DOI:** 10.1371/journal.pone.0109529

**Published:** 2014-10-22

**Authors:** Alessandra Biz, Fernanda Cardoso Farias, Francine Aline Motter, Diogo Henrique de Paula, Peter Richard, Nadia Krieger, David Alexander Mitchell

**Affiliations:** 1 Departamento de Bioquímica e Biologia Molecular, Universidade Federal do Paraná, Curitiba, Paraná, Brazil; 2 VTT Technical Research Centre of Finland, Espoo, Finland; 3 Departamento de Química, Universidade Federal do Paraná, Curitiba, Paraná, Brazil; University of South Florida College of Medicine, United States of America

## Abstract

Recently, it has been suggested that pectinases could be used to hydrolyze pectin in biorefineries based on pectin-rich agro-industrial wastes. However, for this to be viable, the cost of their production would need to be lowered significantly. In fact, over the last few decades, there have been many attempts to improve pectinase production by existing strains or to screen for new strains from environmental isolates. In these studies, it is necessary to measure pectinase activities. Many researchers use single-time-point assays that involve incubation of pectinolytic extracts with pectic substrates for a fixed time, followed by determination of the liberated reducing sugars. However, different researchers use quite different conditions for this assay. Furthermore, no attention has been given to the reaction profile during the assay. In the current work, we show, for the first time, that a significant deceleration of the rate of liberation of reducing sugars occurs over the first ten minutes of the reaction. As a consequence, the incubation time used in a single-time-point assay has a large effect on the value obtained for the activity. In fact, we demonstrate that, depending on the particular combination of incubation time, pectin concentration and reaction temperature, the same extract could be reported to have activities that differ by an order of magnitude. In addition, we show that the relative activities obtained with polygalacturonic acid do not correlate with those obtained with pectin. We conclude that it is currently impossible to make meaningful comparisons between pectinase activities reported in the literature by workers who have used different assay conditions. Therefore there is an urgent need for the development of a standardized assay for evaluating the saccharification potential of pectinase complexes.

## Introduction

The term “pectinases” refers to a group of enzymes that act together to solubilize, de-esterify and depolymerize the complex structure of native pectin [1]. These enzymes have varied applications in the food and beverage industries, including the extraction and clarification of juices, the improvement of the organoleptic properties of wine, the increasing of the efficiency of extraction of vegetable oils and the acceleration of coffee and tea fermentations [2], [3]. Pectinases are also used as additives in animal feeds, mainly for poultry and ruminants, to improve digestibility and nutritional value [4]. Pectinases could also be used for the saccharification of pectin-rich agricultural residues, with the resulting sugars being used for the production of ethanol or platform chemicals [5]–[7]. In this manner, they would play an important role in the recently proposed citrus waste biorefineries [8], [9].

The saccharification of pectin involves a number of enzymes working in conjunction (Table 1) [10], [11]. In the current paper, we use the term “pectinolytic activity” to denote the liberation of reducing sugars from pectin by this mixture of enzymes, which we refer to as a “pectinase complex”. The production of pectinase complexes by submerged-liquid fermentation and solid-state fermentation of various microorganisms has been studied over the last two decades or so, with many researchers having isolated new strains and undertaken medium optimization and strain improvement programs [12], [13]. In these programs, it is necessary to assay pectinolytic activity. When the interest is to produce pectinases for the food industry, it is common to determine pectinolytic activity based on measurements of the reduction in viscosity of a pectin solution [13]. Specific assays based on ruthenium red have also been developed for endopolygalacturoanases [14], [15]. However, the progress towards complete saccharification is best characterized by measuring the liberation of reducing sugars from pectin. The most frequently used method for doing this involves a “single-time-point” assay that requires two steps. The first step is a hydrolysis step, in which the pectinase-containing extract is incubated for a fixed time under selected conditions of pH, temperature and initial pectin concentration. In the second step, the reducing sugars liberated in the first step are quantified, either by the DNS method [16] or by the Somogyi-Nelson method [17], [18]. The advantage of this assay is that it is simple, uses relatively cheap reagents and can be used to process a relatively large number of samples simultaneously. It is performed manually in most laboratories, although a fully automated assay would be possible [19]. Activities are expressed in terms of “µmol of D-galacturonic acid equivalents produced per minute” and reported “per mL of fermentation broth”, in the case of submerged-liquid fermentation, or “per gram of dry solids”, in the case of solid-state fermentation.

**Table 1:** Enzymes that contribute to the degradation of pectin*.

Name	EC number	Mechanism
Pectin lyase	4.2.2.10	Eliminative cleavage of (1→4)-α-d-galacturonan methyl ester to give oligosaccharides with 4-deoxy-6-*O*-methyl-α-d-galact-4-enuronosyl groups at their non-reducing ends
Pectate lyase	4.2.2.2	Eliminative cleavage of (1→4)-α-d-galacturonan to give oligosaccharides with 4-deoxy-α-d-galact-4-enuronosyl groups at their non-reducing ends
Pectate disaccharide lyase	4.2.2.9	Eliminative cleavage of 4-(4-deoxy-α-d-galact-4-enuronosyl)-d-galacturonate from the reducing end of pectate (i.e. de-esterified pectin)
Pectate trisaccharide-lyase	4.2.2.22	Eliminative cleavage of unsaturated trigalacturonate as the major product from the reducing ends of polygalacturonic acid/pectate
Rhamnogalacturonan endolyase	4.2.2.23	Endotype eliminative cleavage of l-α-rhamnopyranosyl-(1→4)-α-d-galactopyranosyluronic acid bonds in rhamnogalacturonan I domains of hairy regions of pectin
Endo-polygalacturonases	3.2.1.15	Random hydrolysis of (1→4)-α-d-galactosiduronic linkages in pectate and other galacturonans
Exo-polygalacturonases	3.2.1.67	Hydrolysis of d-galacturonic acid residues from the reducing ends of polygalacturonate chains
Pectin methyl esterases	3.1.1.11	Demethoxylation of pectin, forming pectate
Pectin acetyl esterases	3.1.1.6	Deacetylation of pectin, forming pectate
Exo-poly-α-galacturonosidase	3.2.1.82	Hydrolysis of pectic acid from the non-reducing end, releasing digalacturonate
Rhamnogalacturonan hydrolase	3.2.1.171	Endohydrolysis of α-d-GalA-(1→2)-α-l-rha-glycosidic bonds in the rhamnogalacturonan I backbone, releasing oligosaccharides with β-d-GalA at the reducing end.
α-l-rhamnosidase	3.2.1.40	Hydrolysis of the glycosidic bonds between rhamnose and galacturonic acid residues
Arabinan endo-1,5-α-l-arabinanase	3.2.1.99	Endohydrolysis of (1→5)-α-arabinofuranosidic linkages in (1→5)-arabinans
Arabinogalactanendo-β-1,4-galactanase	3.2.1.89	Hydrolysis of (1→4)-β-d-galactosidic linkages in type I arabinogalactans.
α-l-arabinofuranidases	3.2.1.55	Hydrolysis of terminal non-reducing α-l-arabinofuranoside residues in α-l-arabinosides.

*Source: [10], [11].

In order to evaluate the success of screening and strain optimization programs, it is necessary to compare the results obtained for pectinolytic activity with those reported in the literature. However, different authors carry out the hydrolysis step quite differently, with either pectin or polygalacturonic acid being used as the substrate and with diverse values being used for the pH, temperature, reaction time and substrate concentration (Table 2) [20]–[36]. These different assay conditions have consequences for comparing results, but these consequences have not yet been explored. Further, although some efforts have been made to measure initial velocities in the characterization of the kinetics of individual enzymes [37], the exact shape of the early reaction profile during assays for the liberation of reducing sugars by pectinase complexes is not clear: the profiles that have been obtained involve measurements that are rather widely spaced in time and initial rates are either not characterized or the profiles themselves not reported [38], [39]. We address these two issues in the current paper, firstly, by obtaining detailed profiles for the liberation of reducing sugars in reactions carried out under different conditions of temperature and initial pectin concentration and, secondly, by comparing reaction profiles for two commercial preparations and two crude extracts, using citric pectin and polygalacturonic acid. We identify, for the first time, the existence of a significant deceleration over the first ten minutes of the reaction and discuss the implication of this early deceleration, and other results, for the assaying of pectinolytic activities.

**Table 2:** Hydrolysis conditions used in assays for pectinase activities reported in the literature.

Microorganism (cultivation mode)*	Pectic substrate & concentration (% m/v)#	T (°C)	t (min)	pH	Activity (SSF: U/g; SLF: U/mL)	Reference
*Thermomucor indicae-seudaticae* (SSF)	1% pectin	60	10	5.5	108	[20]
*Penicillium viridicatum* (SSF)	0.8% pectin	50	10	5.5	71	[21]
*Thermoascus aurantiacus* (SSF)	0.8% pectin	55	10	5.0	43	[22]
*Fusarium moniliforme* (SSF)	0.5% pectin	40	30	4.5	43	[23]
*Aspergillus niger* (SSF)	0.45% pectin	45	30	4.5	25	[24]
*Aspergillus awamori* (SSF)	0.33% pectin	45	10	5.0	9	[25]
*Streptomyces sp.* (SLF)	1% pectin	60	-	3	162	[26]
*Penicillium occitanis* (SLF)	0.45% pectin	50	60	4.8	64	[27]
*Aspergillus niger* (SLF)	0.5% pectin	-	15	4.5	34	[28]
*Aspergillus flavipes* (SLF)	1% pectin	45	20	5	20	[29]
*Aspergillus niger* (SLF)	0.2% pectin	30	-	5.3	1.3	[30]
*Trichoderma viridae* (SlSF)	0.2% PGA	30	-	5	10	[31]
*Aspergillus sojae* (SSF)	0.2% PGA	26	-	6.6	30	[32]
*Bacillus subtilis* (SSF)	0.24% PGA	65	10	9.5	6592	[33]
*Aspergillus niger* (SSF)	-	40	-	3.5	135	[34]
*Aspergillus sp.* (SSF)	0.17% PGA	-	10	5.3	390	[35]
*Penicillium grisereoserum* (SLF)	0.75% PGA	40	20	4.8	3490	[36]

*SSF: solid-state fermentation; SLF: submerged fermentation; SlSF: slurry-state fermentation.

# PGA: polygalacturonic acid.

-: not specified.

## Materials and Methods

### Strains

*Aspergillus niger* CH4 is maintained in the culture collection of the Biomedical Research Institute of the Universidad Nacional Autónoma de México (Mexico City, Mexico) and was kindly provided by Prof. Dr. Jesus Cordova and Prof. Dr. Gustavo Viniegra-Gonzalez. *Aspergillus oryzae* CPQBA 394-12 DRM 01 was isolated from a passion fruit peel and is maintained in the culture collection of the University of Campinas (Campinas, Brazil).

### Substrates for solid-state fermentations

Wheat bran was purchased from the municipal market of Curitiba, located in the state of Paraná, Brazil, and was used as obtained. Sugarcane bagasse was obtained from Destilarias Melhoramentos S/A (Jussara, Brazil) and was sieved to recover particles between 1.7 and 1 mm. Citrus pulp was obtained from the agro-industrial cooperative Corol (Rolândia, Brazil) and was dried to 13% (w/w, wet basis) moisture.

### Preparation of crude extracts from solid-state fermentations

The medium contained 1.5 g dry sugarcane bagasse and 3.5 g of either dry wheat bran (for *A. niger* CH4) or dry citrus pulp (for *A. oryzae* CPQBA 394-12 DRM 01). The mixture was placed in a 250-mL Erlenmeyer flask and autoclaved (121°C, 15 min). Saline solution (containing, in g/L, K_2_HPO_4_ 3, (NH_4_)_2_SO_4_ 13, MgSO_4_.7H_2_O 5, KCl 10, FeSO_4_.7H_2_O 0.09) was autoclaved (121°C, 15 min) and added to each flask to obtain a final moisture content of 70% (w/w, wet basis). The solid media were inoculated with 10^7^ spores per gram of dry substrate and incubated at 30°C for 24 h. The fermented solids were then lyophilized and stored at 4°C. For the extraction, 100 mL of acetate buffer (0.2 M, pH 4.5) was added per gram of lyophilized fermented solids and the mixture was incubated on an orbital shaker at 30°C for 30 min at 180 rpm. The crude extracts were vacuum filtered using Whatman n°1 filter paper. The filtrate was stored at 4°C.

### Commercial enzymes and pectic substrates

The commercial enzyme preparations used were Pectinex Ultra SPL (Novozymes, Denmark), from *Aspergillus aculeatus*, and Pectinase P4716 (Sigma-Aldrich, USA), from *Aspergillus niger*. These preparations were diluted 2500-fold in acetate buffer (0.2 M, pH 4.5) before adding to the substrate mixture. Citric pectin (D-galacturonic acid content>74% and methoxy content>6.7%) and polygalacturonic acid (∼95%, molecular weight 25000–50000), both from Sigma-Aldrich (product numbers P9135 and 81325, respectively), were solubilized in acetate buffer (0.2 M, pH 4.5).

### Determination of the profiles for the release of reducing sugars

The studies of the effect of temperature and pectin concentration on the reaction profile were undertaken using the crude extract of *A. niger* CH4. For this, multiple identical tubes were prepared, each containing 0.25 mL of crude extract and 0.25 mL of citric pectin in acetate buffer (0.2 M, pH 4.5). The stated pectin concentration represents the value for the mixture at zero time. The samples were incubated at the stated temperature. At 30-s intervals, 0.5 mL of DNS was added to the mixture to stop the reaction. All the profiles were obtained with aliquots taken from the same crude extract.

In the study of initial hydrolysis profiles undertaken with different pectinase complexes and pectic substrates, 15 mL of the appropriate substrate solution was incubated in a 100-mL jacketed reactor at 30°C. To start the reaction, 15 mL of the appropriate pectinase preparation was added, giving an initial substrate concentration in the reaction mixture of 0.5% (m/v). Samples of 0.5 mL were taken at intervals of 10 s (0–2 min), 30 s (2–15 min) or 1 min (15–37 min). Each sample was added to 0.5 mL of DNS in an ice bath to stop the reaction.

In the study of long hydrolysis times that was undertaken with different pectinase complexes and pectic substrates, 75 mL of the appropriate substrate solution was incubated in a 250-mL Erlenmeyer flask at 30°C. To start the reaction, 75 mL of the appropriate enzyme preparation was added, giving an initial substrate concentration of 0.5% (m/v). The mixture was incubated on an orbital shaker at 200 rpm and 30°C for 72 h. Samples of 0.5 mL were taken at various times and added to 0.5 mL of DNS in an ice bath to stop the reaction.

The liberation of reducing sugars was followed using the DNS method [16]. Zero time absorbances were subtracted from the readings. Reducing sugar concentrations were estimated as D-galacturonic acid equivalents, using a calibration curve constructed with D-galacturonic acid (Sigma-Aldrich, ≥98.0% purity) concentrations from 0.94 to 9.4 mmol/L.

### Determination of corresponding single-time-point activities from reaction profiles

**Figure 1:**
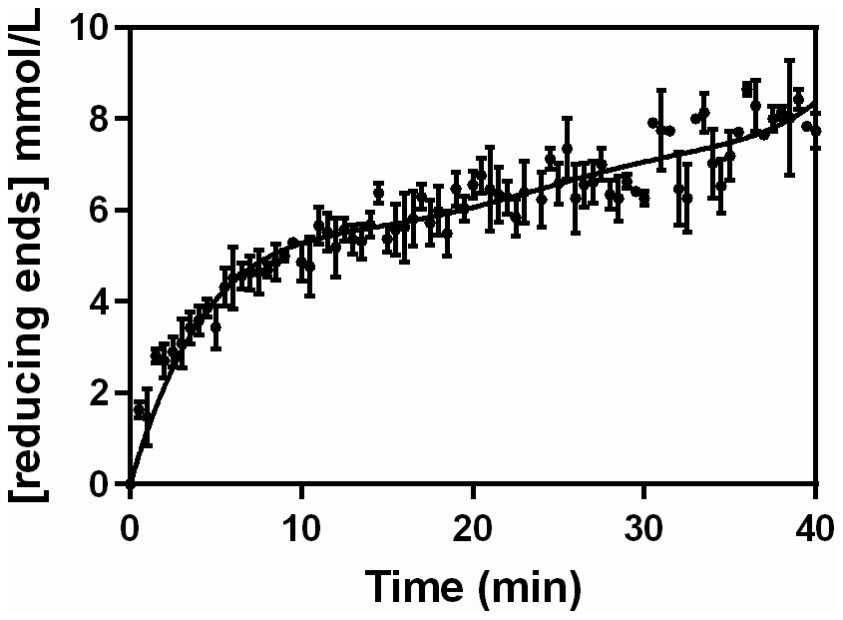
Detailed profile for the hydrolysis of pectin during the first 40 min of the reaction. The hydrolysis was performed at 40°C, with an initial pectin concentration of 0.5% (w/v), using the pectinase complex produced by *Aspergillus niger* CH4 in solid-state fermentation. The reaction was undertaken in triplicate. The mean values for each time were plotted, with the error bars representing the standard error of the mean. The curve represents the best fitting fifth-order polynomial.

A fifth-order polynomial was fitted to the reaction profile obtained under each set of conditions. For all fits, the value of R^2^ was ≥0.90. The fitted curve is only shown in Figure 1; for the sake of clarity, other fitted curves are not shown, since the graphs already contain several different plots. The activity that would have been obtained in a single-time-point assay undertaken using a particular time under a particular set of reaction conditions, denominated *A*(*t*) and with units of U/g, was then calculated as:
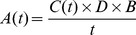
(1)where *C*(*t*) is the reducing sugar concentration (µmol/mL, which is numerically equivalent to the value plotted on the graph as mmol/L) at time *t* (min), obtained by substituting *t* into the corresponding fitted polynomial, *D* is the dilution factor for the extract in the assay (equal to 2) and *B* is the ratio of buffer to dry solids used in the extraction step (mL/g, equal to 100). Note that 1 U corresponds to the production of 1 µmol of D-galacturonic acid equivalents per min.

### Stability assays

The crude extract obtained from solid-state fermentation of *A. niger* CH4 was incubated for 10, 30 and 60 min at 30, 40, 50 and 55°C. The assay for residual activity was undertaken at 50°C, for 10 min, using 0.5% (w/v) pectin (initial concentration in the reaction mixture). The residual activity was calculated relative to the activity obtained in an assay undertaken under the same conditions but without previous incubation.

## Results

### Reaction profiles under different conditions

Since pectin degradation profiles that have been published in the literature typically only provide data points at intervals of several minutes, the first experiment involved the characterization of the degradation of pectin by a crude extract from *A. niger* CH4, using intervals of only 30 s. It was done at 40°C and with a pectin concentration of 0.5% (w/v). A key result, which has not previously been reported in the literature, was that the rate of liberation of reducing sugars decelerated significantly over the first 10 min (Figure 1). Then, from 10 to 40 min, there was an almost linear increase in reducing sugar concentration.

**Figure 2:**
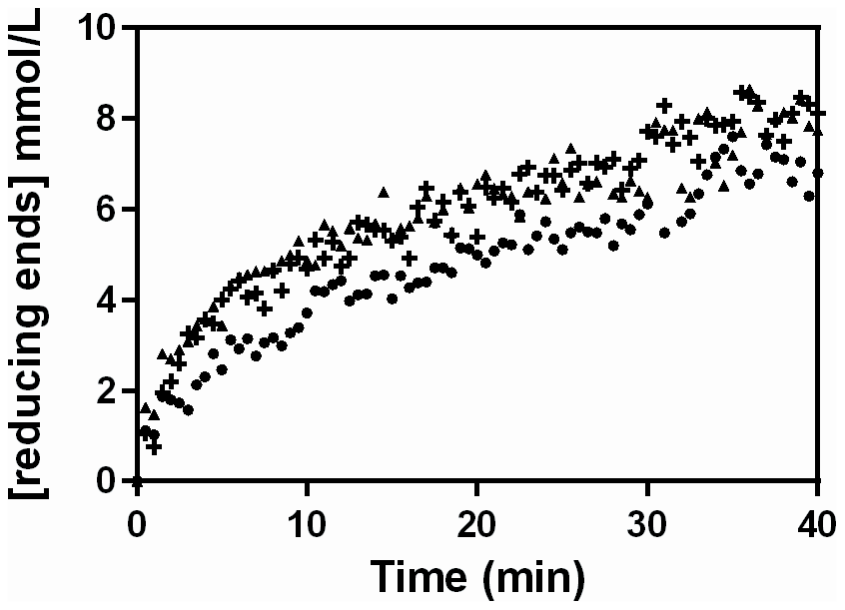
Reaction profiles obtained over the first 40 min at different temperatures. The hydrolysis was performed using 0.5% (m/v) pectin, with the pectinase complex produced by *Aspergillus niger* CH4 in solid-state fermentation. Symbols: (•) 30°C, (▴) 40°C and (**+**) 50°C.

Reaction profiles were also followed at various different temperatures, using a pectin concentration of 0.5% (w/v). As in the previous experiment, in all cases there was a significant deceleration over the first 10 min (Figure 2). The effect of temperature on the liberation of reducing sugars was greatest over this 10 min period, with the amount of reducing sugars at 40 and 50°C being slightly higher than that at 30°C. From 10 min onwards, the profiles had similar slopes.

**Figure 3:**
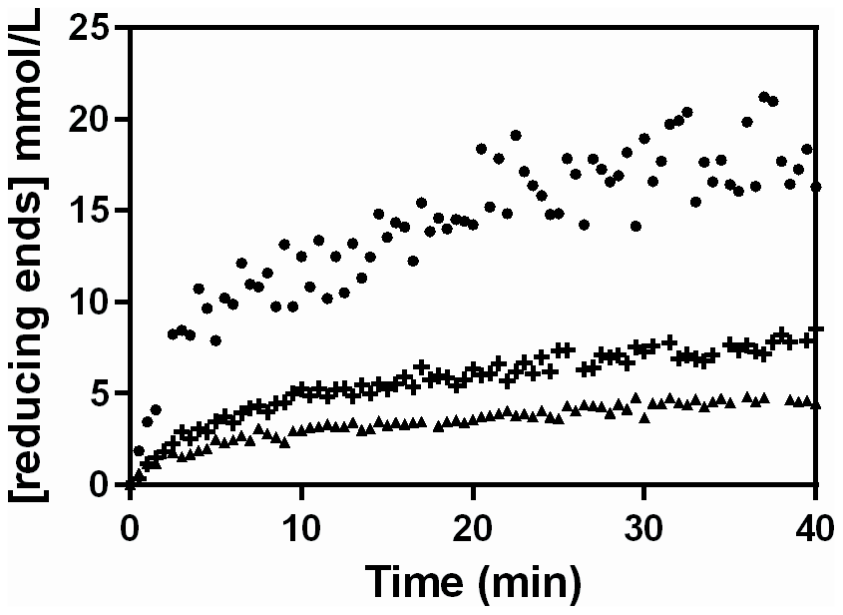
Reaction profiles obtained over the first 40 min with different initial pectin concentrations. The hydrolysis was performed at 45°C, with the pectinase complex produced by *Aspergillus niger* CH4 in solid-state fermentation. Symbols: Initial pectin concentrations of (▴) 0.25% m/v, (**+**) 0.5% m/v and (•) 1% m/v.

Reaction profiles were then followed, at 45°C, with different initial pectin concentrations (Figure 3). The initial reaction rate was highest with 1% (w/v) pectin. However, this concentration led to a viscous solution, which made pipetting difficult, and undissolved pectin was visible suspended in the reaction medium. These problems are probably responsible for the greater dispersion of data points in the 1% (w/v) profile in Figure 3, compared to the profiles obtained at lower initial pectin concentrations. Again, in all the conditions, a significant deceleration occurred over the first 10 min.

### Effect of temperature on loss of pectinolytic activity

**Figure 4:**
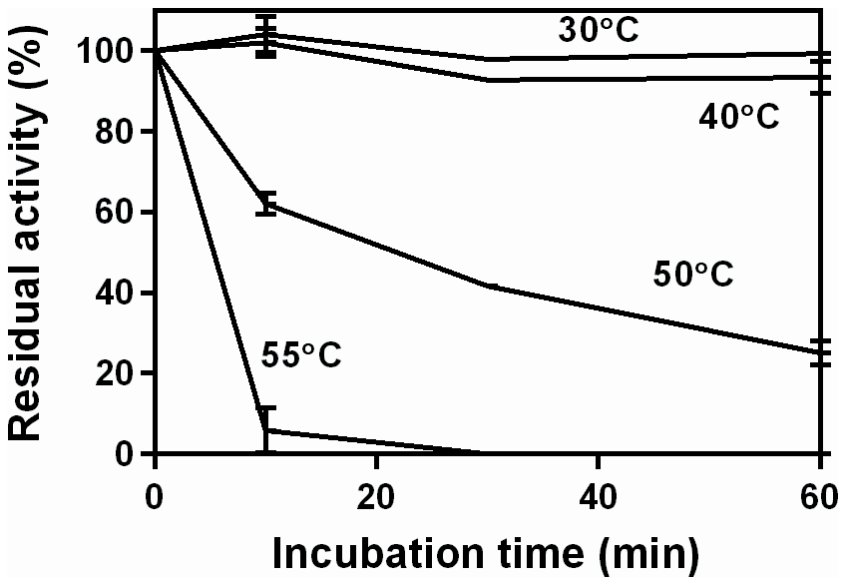
Residual activity curves for the extract incubated over 60 min at different temperatures. The pectinase complex produced by *Aspergillus niger* CH4 in solid-state fermentation was used. Residual activities were obtained at 50°C, in a 10-min assay, using 0.5% pectin (w/v).

Increasing temperatures not only increase the intrinsic rate of enzyme-catalyzed reactions, but also increase the rate of enzyme denaturation. In order to investigate the degree to which denaturation might be occurring in the profiles in Figure 2, the extract was incubated at various temperatures (Figure 4). The pectinase activity was stable over 60 min at 30°C and 40°C. At 50°C, the residual activity fell significantly, reaching only 25% at 60 min. At 55°C, denaturation was significantly faster, with only 6% residual activity at 10 min. These results suggest that thermal denaturation is not the cause of the early deceleration in Figure 1, since that reaction was carried out at 40°C, but that some denaturation probably occurred in the reactions carried out at higher temperatures in Figures 2 and 3.

### Consequences of determining pectinolytic activity using single-time-point assays

The reaction profiles obtained above were used to calculate the pectinolytic activities that would be obtained in single-time-point (STP) assays, carried out under various combinations of temperature and pectin concentration that are typically used in the literature (see Table 2). These will be referred to as “STP-activities”. STP activities were calculated for each of three commonly used reaction times, 10, 15 and 30 min, using the reaction profiles in Figures 2 and 3 (Table 3). It is important to note that all the assays in these two figures involved the same amount of enzyme, with the differences in STP-activity being due to the conditions and the time chosen for the reaction. The STP-activity was influenced most by the reaction time and the concentration of pectin. Importantly, the highest value of STP-activity, obtained for 1.0% (w/v) pectin, 45°C and 10 min, was 8-fold greater than the lowest value of STP-activity, obtained for 0.25% (w/v) pectin, 45°C and 30 min.

**Table 3:** Single-time-point pectinase activities that would be obtained for the same enzymatic extract if incubated under different reaction conditions for different times.

Reaction conditions		“Single-time-point activity” (STP-activity) (U/g) at the stated reaction times*		
[pectin] (%, w/v)	T (°C)	10 min	15 min	30 min
0.25	45	60	44	29
0.5	30	77	59	40
0.5	40	106	76	47
0.5	45	95	72	47
0.5	50	100	73	47
1.0	45	242	176	119

*Calculated from the data in Figures 2 and 3 using Eq. (1).

### Early profiles for different pectinase preparations acting on different pectic substrates

**Figure 5:**
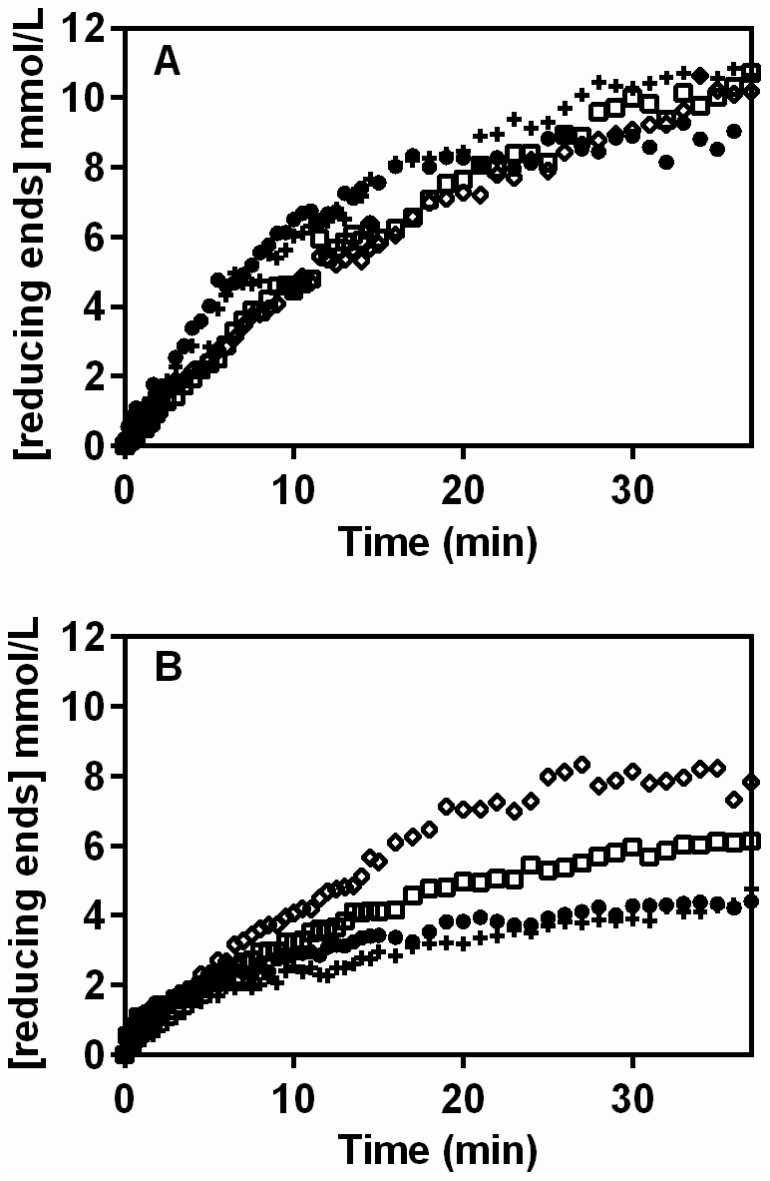
Short hydrolysis profiles (40 min), obtained using different pectinase complexes and either polygalacturonic acid (A) or citric pectin (B). The hydrolysis was performed at 40°C, with an initial substrate concentration of 0.5% (w/v). The pectinase complexes used were the extracts of *Aspergillus niger* (•) and *Aspergillus oryzae* (+), produced in solid-state fermentation, and the commercially available Pectinex Ultra SPL (Novozymes) (□) and Pectinase P4716 (Sigma-Aldrich) (⋄).

To evaluate how the type of pectic substrate affects the activity, profiles for the liberation of reducing sugars were obtained for both polygalacturonic acid and citric pectin. Based on the results in Figures 2 to 4, the conditions chosen for the reactions were 0.5% (m/v) pectic substrate and 30°C. Profiles were obtained with four different pectinase complexes: a crude extract from *A. niger* CH4 (maintained as a reference), a crude extract from *A. oryzae*, Pectinase P4716 and Pectinex Ultra SPL. These pectinase complexes were diluted so as to give similar profiles on polygalacturonic acid (Figure 5A). The same dilutions were then used for the degradation of citric pectin (Figure 5B).

Three key observations can be made. First, all profiles in Figures 5A and 5B show the early deceleration observed in Figures 1, 2 and 3. Second, comparing the same pectinase complex on different substrates, the rate of liberation of reducing sugars from citric pectin was lower than that obtained with polygalacturonic acid. Third, comparing different pectinase complexes on the same substrate, although the amounts of these complexes were chosen so as to give similar hydrolysis profiles with polygalacturonic acid, the profiles obtained were quite different from one another when these same amounts were added to citric pectin. The most effective hydrolysis of citric pectin was obtained with Pectinase P4716.

### Extended profiles for different pectinase preparations acting on different pectic substrates

**Figure 6:**
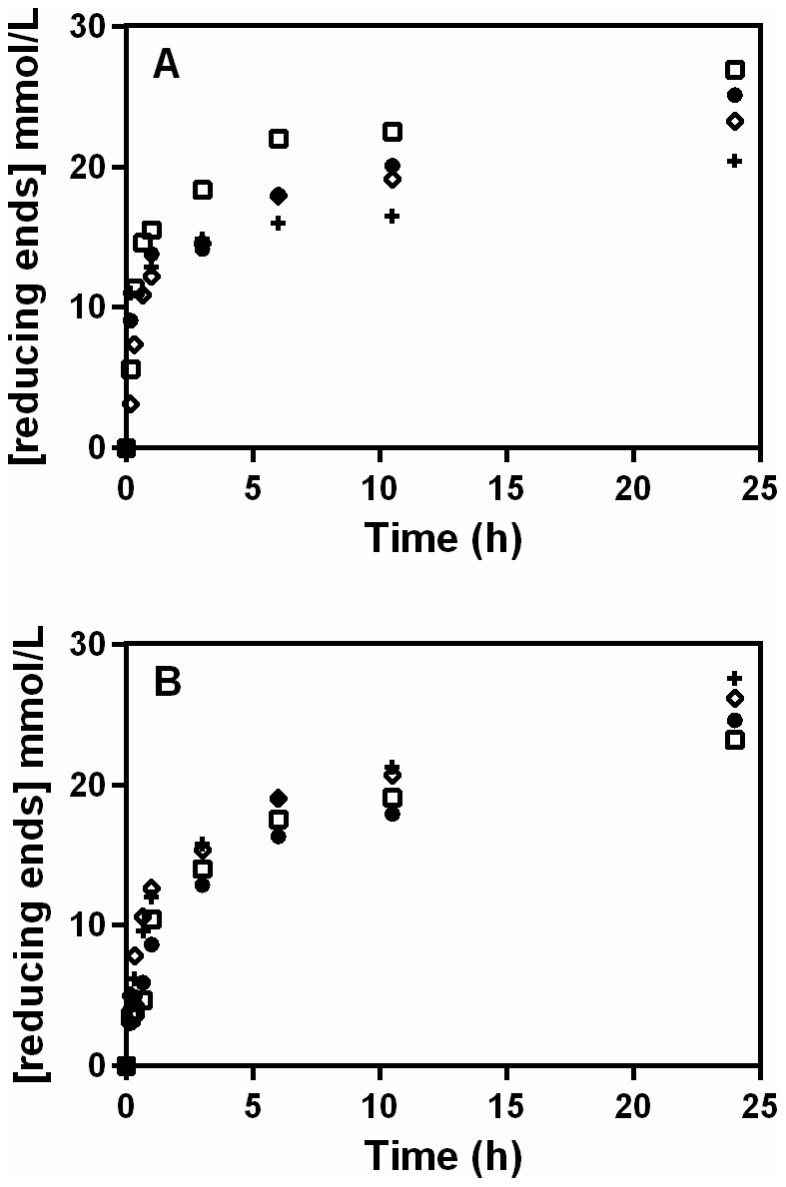
Extended hydrolysis profiles (24 h), obtained using different pectinase complexes and either polygalacturonic acid (A) or citric pectin (B). The hydrolysis was performed at 40°C, with an initial substrate concentration of 0.5% (w/v). The pectinase complexes used were the extracts of *Aspergillus niger* (•) and *Aspergillus oryzae* (**+**), produced in solid-state fermentation, and the commercially available Pectinex Ultra SPL (Novozymes) (□) and Pectinase P4716 (Sigma-Aldrich) (⋄).

The same amounts of pectinase complex that were used for the hydrolysis of polygalacturonic acid in Figure 5A were added to 0.5% (m/v) of the two pectic substrates and the reactions were followed for 72 h at 30°C. The results are plotted for 24 h since in all cases there was no further liberation of reducing sugars after this time (Figures 6A and 6B).

Two key observations can be made. First, there was a continued deceleration of the reaction up to 6 h, but this deceleration was less pronounced than that which occurred over the first 10 min in Figures 1, 2, 3 and 5. This was followed by a slow, essentially constant rate of reducing sugar release from 6 to 24 h. Second, although Pectinase P4716 liberated significantly more reducing sugars from citric pectin than did the other complexes over the first 40 min of reaction (Figure 5B), this superior efficiency was not maintained. In fact, at 24 h, slightly more reducing sugars had been liberated by the extract from *A. oryzae* than by Pectinase P4716.

## Discussion

Our work makes two main contributions. First, we have demonstrated that, during the hydrolysis of polygalacturonic acid and citric pectin by pectinase complexes from various sources, there is a significant deceleration of the rate of release of reducing sugars over the first ten minutes, followed by a longer, slower, deceleration over several hours. Second, we have identified several important issues with respect to the single-time-point assays that are commonly used for determining pectinase activities. These contributions are discussed below.

### Implications of the early deceleration

Although the release of reducing sugars from pectic substrates by pectinase complexes has already been studied [38]–[40], in these studies the reaction profiles were obtained by taking samples at intervals of up to ten minutes or more. By removing samples at 30-s intervals, we have, for the first time, demonstrated that there is a significant deceleration in the rate of release of reducing sugars over the first ten minutes of reaction.

This early deceleration has important consequences for the determination of pectinase activities by measuring the liberation of reducing sugars in STP-assays. As Table 3 shows, for the same pectinase preparation and for any given set of reaction conditions, the activity that would be determined in an STP-assay decreases significantly as the incubation time is increased. This occurs because increases in reaction time above 10 min are associated with proportionally small increases in the reducing sugar level, so the result given by Eq. (1) becomes increasingly smaller.

Interestingly, the early deceleration is not apparent in the profiles that Baciu and Jördening [41] presented for the release of D-galacturonic acid from pectin by a different Pectinex product (Pectinex 100 L, Novozymes). Their profiles cover the first 300 min and show a gradual deceleration over this time. Our experiments differ from theirs in an important aspect: we measured the liberation of reducing sugars and therefore detected not only D-galacturonic acid in itself, but also oligomers produced by endo-acting enzymes. Baciu and Jördening attributed the gradual deceleration that occurred in their studies to product inhibition by D-galacturonic acid [41]. This is likely to be the cause of the slow deceleration that occurred over longer hydrolysis times in the current work (see Figure 6). However, further research is required to determine whether the early deceleration of the rate of liberation of reducing sugars is also caused by product inhibition.

### Other issues related to the use of single-time-point assays for determining pectinase activities

As Table 3 shows, in STP-assays, the same pectinase preparation could be reported as having a pectinolytic activity ranging over almost an order of magnitude, depending on the particular set of hydrolysis conditions that is chosen. Since previous researchers have used not only different reaction times in carrying out STP-assays for pectinase activity, but also different temperatures and pectin concentrations (Table 2), it is impossible to draw meaningful conclusions about which result from the literature actually represents the best pectinase production. A lower amount of pectinases, assayed under favorable conditions, could lead to an activity higher than that obtained for a higher amount of pectinases, assayed under unfavorable conditions.

This raises an important question for researchers interested in using pectin-rich agro-industrial wastes in biorefineries. One of the processing steps in such biorefineries is likely to be the saccharification of pectin, for which it is desirable to identify good pectinase-producing microorganisms: Is it possible to establish a standard assay for screening pectinase activities that will not only reflect the saccharifying potential of a pectinase preparation, but can also be used to enable direct comparisons with the results of other research groups? It is useful to compare the current situation with respect to pectinase activity determination with the situation of cellulase activity determination in the 1980's. At that time there was much interest in the production of ethanol from cellulosic biomass, but different investigators used different assays and different conditions to determine cellulase activities. Then, in 1987, Ghose [42] proposed standardized methods for the determination of cellulase activity. Most workers in the field of cellulases now use these methods, allowing meaningful comparisons to be made within the cellulase literature.

Some authors have used polygalacturonic acid as the substrate in assays for pectinolytic activity [36], [40], [43], [44]. On the basis of our results, we recommend that, in any assay that aims to evaluate the potential for pectinase preparations to saccharify pectin, pectin should be used as the substrate and not polygalacturonic acid: the activities obtained with polygalacturonic acid are higher than those obtained with pectin, so the use of polygalacturonic acid would lead to an overestimation of activities. Furthermore, the pectinase complex that hydrolyzes polygalacturonic acid most efficiently might not be the pectinase complex that hydrolyzes pectin most efficiently.

It would be a relatively simple matter to select appropriate values for pectin concentration, temperature and pH for a standard pectinase assay based on the liberation of reducing sugars. The initial pectin concentration in the reaction mixture should be as high as possible without causing problems with viscosity (e.g. 0.5% w/v). With respect to temperature, Table 2 shows that various authors are performing activity assays at temperatures of 50°C or more, but a temperature should be chosen that does not cause denaturation (e.g. a value in the range of 30 to 40°C). Temperatures above 50°C lead to high activity values over the short period of the assay, but they are unlikely to be used in saccharification processes of up to 24 h or more, unless the enzymes in the preparation are thermostable. The effect of pH on pectinase activity was not studied in the current work because it has already been well studied [38], [39], [45], [46]. Most pectinases have a pH optimum around 4.5. However, some pectinases, principally those produced by species of *Bacillus*, have a pH optimum between 8 and 10.5 [47], [48].

On the other hand, it is not clear what would be an appropriate reaction time for a standard pectinase assay. Several possibilities might be considered. For example, initial velocities could be determined. However, due to the non-linearity of the reaction profile, it would be necessary to obtain various data points over the first minutes of the reaction in order to be able to estimate the initial velocity with an acceptable precision. In any case, since the greater part of the saccharification process occurs over many hours, after a significant initial deceleration, initial velocities will not necessarily reflect the long-term saccharification potential.

Of course, assays could be carried out over 24 h. In this case, it would be unwise to use a single time point at 24 h, since the reaction might reach completion before this time. It would be advisable, therefore, to have various measurements during this time. However, such a long reaction time would be inconvenient in screening programs, as it would greatly reduce throughput. Researchers are likely to prefer to use such long reaction times in monitoring process development, not in initial screening programs.

One possibility might be to use a “double-time-point” assay. A first reading could be taken after the initial deceleration, at 20 min or so. A second reading could then be taken later, maybe at 40 min or even at 60 min. The activity could then be expressed on the basis of the difference between these two readings. This would not be as convenient as an STP-assay, but would have the advantage of not including the period during which the early deceleration occurs. However, it remains to be proven whether activities obtained in this manner would reflect the long-term saccharification potential.

## Conclusions

We have shown, for the first time, that there is a significant early deceleration in the liberation of reducing sugars from pectic substrates by pectinase complexes. On the basis of this phenomenon, as well as results regarding the effects of temperature and substrate concentration on the reaction profile, we have shown that it is not possible to make meaningful comparisons between pectinase activities reported by different authors who have used different assay conditions, substrates and reaction times in single-time-point assays. There is an urgent need for the establishment of a standardized assay procedure for evaluating the saccharification potential of pectinase complexes.
